# Ontario Veterinary College

**Published:** 1900-05

**Authors:** 


### ONTARIO VETERINARY COLLEGE.

The closing exercises of session 1899–1900 of this institution were held in the college buildings on Thursday, March 29. There was a large gathering of students anxious to hear the results of the recent examinations, and who manifested their interest in the proceedings by frequent bursts of applause as the names of the successful prize-winners were announced. Professor A. Smith, the principal, took the chair, and with him on the platform were Professor Baker, of Toronto University; Mr. H. J. Hill, Industrial Exhibition Association; Dr. Duncan, Dr. Chambers, Professor J. H. Reed, V.S., of Guelph Agricultural College.

Professor Smith opened the meeting by a short address, and called on Dr. Duncan to read over the graduating list, and also the list of prize-winners. The prize-winners were then called forward, and the prizes, consisting of gold and silver medals and professional books, were duly presented. Mr. Hill presented the gold medal given for the best dissected specimen by the Toronto Industrial Exhibition Association.

Professor J. H. Reed, V.S., on behalf of Mr. Wilson, President of the Ontario Veterinary Association, presented the gold medal donated by the association for the best general examination.

Professor Baker gave a short but very interesting address to the students, in which he noted the changes that have prevailed as to the use of the horse for military purposes. A few years ago it was predicted that the perfection of artillery would supersede the horse to a great extent, except for scouting. It is now being demonstrated that the necessity for greater mobility in infantry will require a larger number of horses for mounted infantry. Also for civil purposes it has been predicted that electricity would to a great extent supersede the horse as a motive power; but he confidently believed that the horse for rapid movements would still maintain its old-time ascendancy. He also contended that for humanitarian reasons, for the relief of pain and the treatment of the ailments of all dumb animals, the field for the educated veterinarian would increase and not diminish.

Mr. G. A. Shaw, President of the graduating class, in a short address presented Professor Smith on behalf of the class with a magnificent picture of the class. Professor Smith, in an eloquent address in response, thanked the class most heartily for this indication of the good feeling existing in the college. Thanks were also tendered Professor Baker for his address.

The following is a list of graduates and prize-winners :

G. Harry Acres, Calgary, N. W. T. ; Freeman J. Aldous, Fenelon Falls, Ont.

William P. Barnes, Brookline, Mass.

James A. Campbell, Bedford, England ; W. E. Clemons, Grandville, Ohio.

Henry James Daniels, North Adams, Mass. ; Albert L. Deal, Wilmot, Ohio ; John H. Dellenberger, Jr., Akron, Ohio ; Dolph S. De Wolf, Grand Rapids, Mich. ; Burt Kimball Dow, Cabot, Vermont.

Frank E. Erwin, Fisher, Minn.

Jacob Palmer Foster, Bangor, South Dakota ; Robert Frame, Treherne, Man. ; Joseph Freeman, Hull, England.

Henry James Hagerty, Dubuque, Iowa ; Charles A. Hamilton, Kingston, Ont. ; Oscar Hartnagl, Victoria, B. C. ; L. Morey Holmes, New Orleans, La.

Lewis N. Jargo, Stoughton, Wis. ; Henry N. Jeffries, Greensburg, Ind. ; George L. Jermyn, Wiarton, Ont.

David S. Krull, Clarence Centre, N. Y.

Walter Keys Lewis, Anderson, South Carolina ; Ernest E. Linton, Kingston, Jamaica, W. I. ; James Lucas, Liverpool, England ; Ashley T. Lyster, Richmond, Quebec.

Milton P. McClellan, St. Thomas, Ont. ; James McFadyean, Waldemar, Ont. ; Charles D. McGilvray, Binscarth, Man. ; John D. McLeod, Kincardine, Ont. ; Charles H. McVeigh, Vars, Ont.

Charles Henry Paquin, Barre, Mass.

Arthur C. Ramsay, Exeter, Ont. ; R. S. Reece, St. Ann, Jamaica, W. I. ; John Arthur Rennie, Stevenspoint, Wis. ; Beale Atwood Robinson, Parsons, Kansas ; William T. Rodgers, Toronto, Ont.

George A. Shaw, Manchester, N. Y. ; Edward J. Shirley, Watford, Ont. ; A. Fraser Smith, Claude, Ont. ; George O. Smith, Ligonier, Ind. ; John Macdonald Smith, Kingston, Ont. ; Archibald H. Stewart, Dalesville, Ind. ; H. R. Stewart, Toronto, Ont.

Jacob Van Hont, Heron, N. Y.

Stephen A. K. White, Ottawa, Ont.

